# In Situ Visual Detection of TelMV, EAPV, and PaMoV in Passionfruit Using Reverse Transcription-Recombinase-Aided Amplification and CRISPR/Cas12a

**DOI:** 10.3390/plants15060853

**Published:** 2026-03-10

**Authors:** Cuiping Mo, Youcong Li, Jinqing Chen, Lihui Liu, Lixian Cui, Bixia Qin, Jianhe Cai, Huiting Xie, Zhanbiao Li

**Affiliations:** 1 Plant Protection Research Institute, Guangxi Academy of Agricultural Sciences, Nanning 530007, China; cuiping2018@126.com (C.M.); youge1942@outlook.com (Y.L.); jinqingchen@gmail.com (J.C.); yangmeiliu@yeah.net (L.L.); lixiancui@163.com (L.C.); qinbx@126.com (B.Q.); caijianhe@gxaas.net (J.C.); 2 Key Laboratory of Green Prevention and Control on Fruits and Vegetables in South China, Ministry of Agriculture and Rural Affairs, Nanning 530007, China; 3 Guangxi Key Laboratory of Biology for Crop Diseases and Insect Pests, Nanning 530007, China

**Keywords:** RT-RAA, CRISPR/Cas12a, TelMV, EAPV, PaMoV, visual detection, in situ detection

## Abstract

As a tropical fruit of considerable economic importance, passionfruit (*Passiflora edulis Sims*) is extensively cultivated in the tropical and subtropical regions of China; however, the widespread incidence of viral diseases has significantly hampered the safety of its production. Rapid, sensitive, and visual detection of plant viruses is essential to effectively prevent and manage these viral diseases. In this study, we developed a visual detection system (displaying under blue light or UV) utilizing reverse transcription recombinase-aided amplification (RT-RAA) in conjunction with CRISPR/Cas12a to detect three viruses harmful to passionfruit production: telosma mosaic virus (TelMV), East Asian passiflora virus (EAPV), and passiflora mottle virus (PaMoV). Within this system, the optimal primer concentration for the RT-RAA reaction was determined to be 0.4 μM for all three viruses, with an optimal reaction temperature of 37 °C. The optimal reaction times were established as 20 min for TelMV, 15 min for EAPV, and 30 min for PaMoV. The entire detection process could be completed within 30 min without the need for sophisticated equipment or instruments. For TelMV and EAPV, the detection system demonstrated the capability to detect samples at a dilution of 10^6^, representing an approximately 10^4^-fold improvement over RT-PCR, while for PaMoV, it could identify samples at a dilution of 10^6^, representing an approximately 10^2^-fold improvement over traditional RT-PCR methods. These results confirm the successful development of the CRISPR/Cas12a-based detection systems. Subsequently, the system was applied for in situ detection of the three target viruses in field settings, yielding results that were fully consistent with laboratory-based RT-PCR assays, a consistency which underscores the system’s strong potential for field application in detecting important crop viruses.

## 1. Introduction

As a tropical fruit of considerable economic importance, passionfruit (*Passiflora edulis Sims*) is extensively cultivated in the tropical and subtropical regions of China, including Guangxi, Guangdong, Fujian, Hainan, Yunnan, and Taiwan. In recent years, the passionfruit industry has expanded continuously due to passionfruit’s unique flavor and high market value; however, the widespread occurrence of viral diseases has severely hindered the safety of its production. To date, over 44 viruses have been reported to infect passionfruit [1], with the most significant threats posed by potyviruses such as the telosma mosaic virus (TelMV), east Asian passiflora virus (EAPV), and passiflora mottle virus (PaMoV). Previous investigations by our team have identified TelMV and EAPV as the primary viral pathogens affecting passionfruit in Guangxi [2]. Viral diseases have also been reported in other provinces, with the primarily viral pathogens affecting passionfruit being TelMV, EAPV, and cucumber mosaic virus (CMV) in Fujian [3]; TelMV, PaMoV, EAPV, and CMV in Hainan [1]; and EAPV and TelMV in Guizhou [4]. This highlights the urgent need for a rapid, in situ detection method to facilitate effective viral disease management.

Current detection techniques for plant viruses include biological, serological, and molecular biological approaches. Among the molecular methods, those that are frequently applied include polymerase chain reaction (PCR), reverse transcription–polymerase chain reaction (RT-PCR), loop-mediated isothermal amplification (LAMP and RT-LAMP), molecular hybridization, and enzyme-linked immunosorbent assay (ELISA) [5]. PCR/RT-PCR-based methods, particularly qPCR and RT-qPCR detection methods, are the most widely utilized due to their high sensitivity and specificity. Nevertheless, these techniques necessitate well-equipped laboratories and relatively costly reagents, thereby restricting their application in resource-limited areas [6]. Many of these methods are hindered by challenges such as high cost, limited accuracy, insufficient sensitivity, and the inability to perform in situ diagnosis in resource-poor environments. Thus, establishing rapid, sensitive, and portable virus detection techniques will be essential to advancing the research, prevention, and control of plant viral diseases.

The clustered regularly inter-spaced short palindromic repeat-associated (CRISPR-Cas) systems are sequence-specific RNA-guided endonuclease complexes with the ability to bind and cleave nucleic acids [7]. Over the past decade, CRISPR-Cas technology has been widely applied in eukaryotic organisms for purposes such as genome engineering, molecular immunology, and transcriptional regulation [8,9,10,11,12]. Notably, certain CRISPR-Cas systems, including Cas12 and Cas13, demonstrate collateral cleavage activity upon target recognition, leading to the non-specific degradation of single-stranded DNA (ssDNA) or RNA reporters [13,14]. This feature has been utilized for creating highly sensitive nucleic acid detection platforms, such as SHERLOCK (Specific High-sensitivity Enzymatic Reporter UnLOCKing), HOLMES (one-Hour Low-cost Multipurpose highly Efficient System), and DETECTR (DNA Endonuclease-Targeted CRISPR Trans Reporter) [15,16,17,18,19], which offer high sensitivity and visual readouts. Isothermal amplification techniques, such as loop-mediated isothermal amplification (LAMP) and recombinase-aided/polymerase amplification (RAA/RPA), facilitate nucleic acid amplification at a constant temperature, making them suitable for in situ diagnosis in resource-limited environments [20,21]. When combined with reverse transcriptase, LAMP and RAA are transformed into RT-LAMP and RT-RAA, respectively, enabling their application in the detection of RNA viruses [22]. For instance, a visual detection method for prunus necrotic ring-spot virus (PNRSV) was established by combining RT-RAA with CRISPR/Cas12a, providing a convenient and efficient assay for field diagnosis and prediction of PNRSV in China [23]. Similarly, an RT-RAA-CRISPR/Cas12a assay was developed for tomato mottle mosaic virus (TMMV) detection, providing a more convenient technical tool for field and portable diagnosis in China [24].

TelMV, EAPV, and PaMoV are three viral pathogens that significantly impact passionfruit production in south China. The precise detection of these viruses is essential for ensuring the safe production of passionfruit seedlings and fruit in this region. Consequently, in this study we have developed a rapid, sensitive, specific, and visual detection method utilizing RT-RAA and CRISPR/Cas12a systems to identify TelMV, EAPV, and PaMoV, respectively. These methods provide a more convenient technical tool for the field diagnosis and forecasting of passionfruit viral diseases, and they hold substantial significance for the prevention and control of such diseases in China.

## 2. Results

### 2.1. Optimization of RT-RAA Conditions

**Figure 1:**
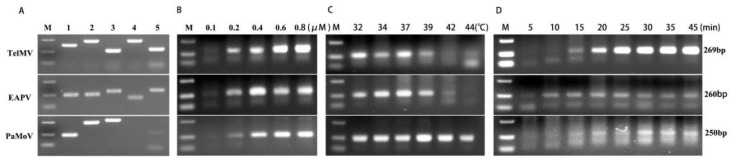
Selection and optimization of RT-RAA detection primers for TelMV, EAPV, and PaMoV. (**A**) Screening of primers for TelMV, EAPV, and PaMoV RT-RAA; (**B**) screening of primer concentrations for TelMV, EAPV, and PaMoV RT-RAA; (**C**) screening of reaction temperatures for TelMV, EAPV, and PaMoV RT-RAA; and (**D**) screening of reaction times for TelMV, EAPV, and PaMoV RT-RAA.

In this study, we optimized the RT-RAA reaction conditions by using the total RNA extracted from passionfruit leaf samples infected with TelMV, EAPV, and PaMoV. Multiple RT-RAA primer sets, with five pairs of primers for each virus, were designed based on the conserved region of the TelMV, EAPV, and PaMoV genome sequences (Supplementary Table S1). Among these five primer sets, the most active ones were selected for subsequent experiments: RAA-TelMV-3F/R (269 bp), RAA-EAPV-1F/R (260 bp), and RAA-PaMoV-1F/R (250 bp) (Figure 1A). We then evaluated the effects of different primer concentrations, reaction temperatures, and reaction times on RT-RAA efficiency. As shown in Figure 1B, optimal amplification results were achieved for all three viruses when the primer concentration ranged from 0.2 to 0.8 μM; therefore, a primer concentration of 0.4 μM was selected for all three viruses (Figure 1B). A reaction temperature range of 32–39 °C yielded satisfactory amplification results, and so 37 °C was ultimately determined as the optimal RT-RAA reaction temperature (Figure 1C). Similarly, a reaction time range of 15–45 min produced good amplification results, so after comprehensive consideration, the RT-RAA reaction times were set at 20 min for TelMV, 15 min for EAPV, and 30 min for PaMoV (Figure 1D).

### 2.2. Validation of CRISPR/Cas12a Rapid Detection System Activity

**Figure 2:**
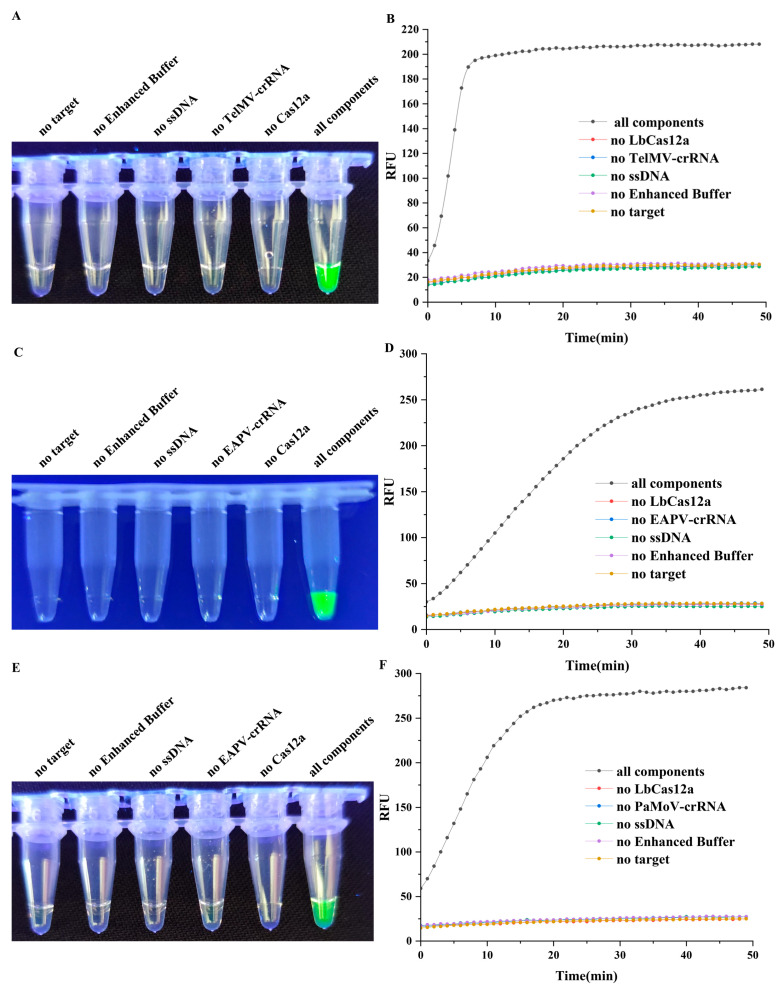
The activity validation of CRISPR/Cas12a assay system using TelMV, EAPV, and PaMoV positive standard plasmid. (**A**,**C**,**E**): Fluorescence status observed under blue light irradiation after 50 min reaction at 37 °C with different component deletions; (**B**,**D**,**F**): Real-time fluorescence change trends during 50 min reaction at 37 °C with different component deletions.

To verify the feasibility of the CRISPR/Cas12a rapid detection system, we confirmed the necessity of each reaction component and the activity of the LbCas12a protein. The positive standard plasmids of pEASY^®^-Blunt Zero-TelMV, pEASY^®^-Blunt Zero-EAPV, and pEASY^®^-Blunt Zero-PaMoV were constructed and used as the substrates for the detection system. After amplification, the RT-RAA products were subjected to a CRISPR/Cas12a detection assay. When the RT-RAA amplicons were recognized by its specific crRNA and formed a complex with LbCas12a, the activated LbCas12a cleaves a quenched green fluorescent ssDNA reporter, generating a strong green fluorescence signal. To assess the role of each component, the detection reactions were performed separately, with each omitting one of the following: the LbCas12a protein, crRNA (TelMV-crRNA, EAPV-crRNA, or PaMoV-crRNA), ssDNA reporter, 10× Enhanced Buffer, or RT-RAA product. The reactions were carried out at 37 °C for 50 min in a multi-functional microplate reader, with fluorescence intensity recorded every minute. After incubation, the reaction tubes were observed under blue light with the resulting showing that the fluorescence signal intensity in reaction tubes increased rapidly when pEASY^®^-Blunt Zero-TelMV was used as the substrate, reaching nearly maximum intensity within 10 min (Figure 2A,B). For pEASY^®^-Blunt Zero-EAPV, the fluorescence signal continued to rise after 30 min but at a markedly slow rate (Figure 2C,D), while for pEASY^®^-Blunt Zero-PaMoV, the fluorescence signal essentially plateaued after 20 min (Figure 2E,F). In contrast, no fluorescence signal was observed in reactions lacking LbCas12a, crRNA, ssDNA, RT-RAA product, or 10× Enhanced Buffer; only tubes containing all components exhibited strong green fluorescence. These results indicated that the CRISPR/Cas12a detection system was feasible and that every component in the reaction system was indispensable.

### 2.3. Analysis of the Specificity the RT-RAA-CRISPR/Cas12a Detection Method

**Figure 3:**
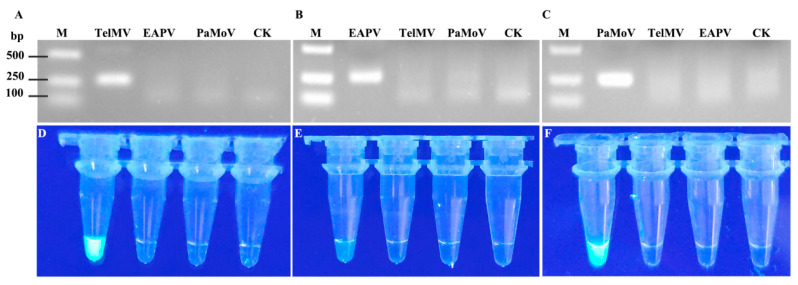
Validation of the specificity of RT-RAA-CRISPR/Cas12a assays for TelMV, EAPV, and PaMoV using positive standard plasmids. M: D2000 Plus DNA Maker; (**A**–**C**): RT-PCR detection results of primer pairs RAA-TelMV-3F/R, RAA-EAPV-1F/R, and RAA-PaMoV-1F/R for three target viruses; CK: the water control; and (**D**–**F**): Visual detection (displaying under blue light) results of primer pairs RAA-TelMV-3F/R, RAA-EAPV-1F/R, and RAA-PaMoV-1F/R for three target viruses using RT-RAA-CRISPR/Cas12a.

TelMV, EAPV, and PaMoV all belong to the genus Potato Y virus, and their genome sequences share high similarity. To further validate the specificity of the primers RAA-TelMV-3F/R, RAA-EAPV-1F/R, and RAA-PaMoV-1F/R, RT-RAA reactions were performed using pEASY^®^-Blunt Zero-TelMV, pEASY^®^-Blunt Zero-EAPV, and pEASY^®^-Blunt Zero-PaMoV positive standard plasmids as templates. After the reactions, 0.8 μL of the amplicons were collected for CRISPR/Cas12a detection, while the remaining RT-RAA reaction products were purified using a PCR purification kit and subjected to 2% agarose gel electrophoresis. The results demonstrated that the electrophoretic band target was only detected for the corresponding target viruses (Figure 3A–C) and specific fluorescence signals were observed (Figure 3D–F). These data indicate that the primers RAA-TelMV-3F/R, RAA-EAPV-1F/R, and RAA-PaMoV-1F/R primers exhibit good specificity and the developed RT-RAA-CRISPR/Cas12a visual rapid detection method shows excellent detection specificity.

### 2.4. Analysis of the Sensitivity the RT-RAA-CRISPR/Cas12a and RT-PCR

**Figure 4:**
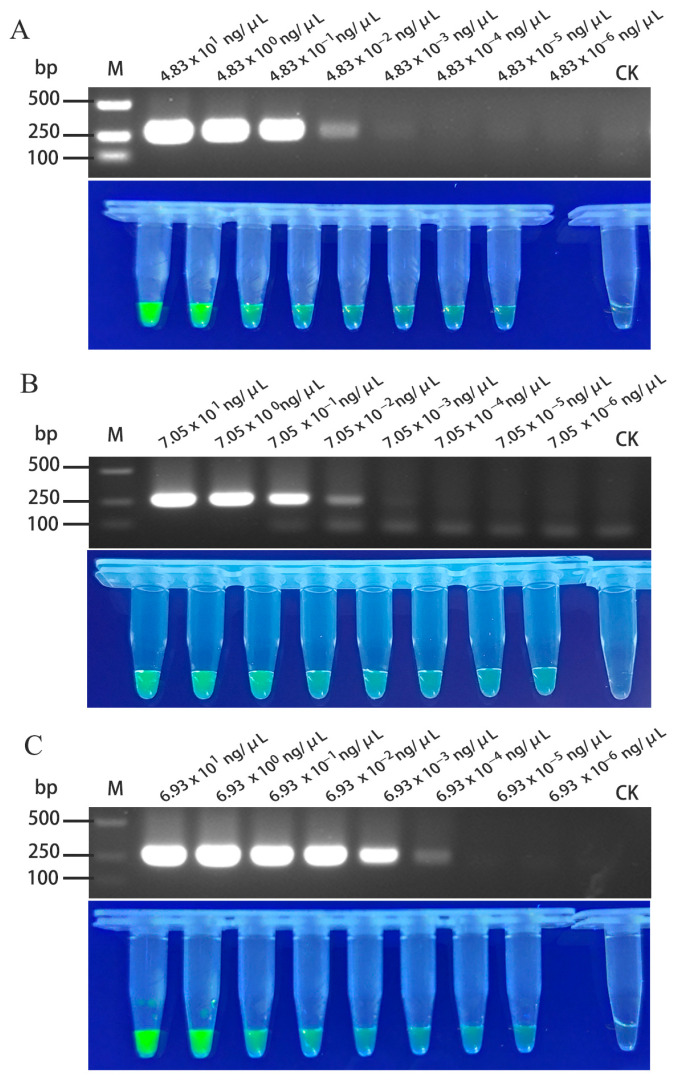
Comparative analysis of the detection sensitivity of RT-PCR and RT-RAA-CRISPR/Cas12a for TelMV, EAPV and PaMoV. (**A**) Comparative analysis of the detection sensitivity of RT-PCR and RT-RAA-CRISPR/Cas12a for TelMV at different dilution factors; (**B**) comparative analysis of the detection sensitivity of RT-PCR and RT-RAA-CRISPR/Cas12a for EAPV at different dilution factors; and (**C**) comparative analysis of the detection sensitivity of RT-PCR and RT-RAA-CRISPR/Cas12a for PaMoV at different dilution factors.

The total RNA extracted from passionfruit leaves infected with TelMV, EAPV, and PaMoV was used as a template, with initial concentrations of 4.83 × 10^1^ ng/μL, 7.05 × 10^1^ ng/μL, and 6.93 × 10^1^ ng/μL, respectively. Following serial dilution, the samples were detected by both conventional RT-PCR and the RT-RAA-CRISPR/Cas12a detection method to compare their sensitivity. The results showed that RT-PCR could detect TelMV at a 10^4^-fold dilution (4.83 × 10^−3^ ng/μL), while the RT-RAA-CRISPR/Cas12a method remained effective at a 10^7^-fold dilution (4.83 × 10^−6^ ng/μL), indicating a 10^4^-fold-higher sensitivity than conventional RT-PCR (Figure 4A). For EAPV, the RT-PCR detection was effective up to a 10^4^-fold dilution (7.05 × 10^−3^ ng/μL), whereas the RT-RAA-CRISPR/Cas12a method still produced a clear green fluorescence signal at 10^7^-fold dilution (7.05 × 10^−6^ ng/μL), corresponding to a 10^4^-fold improvement in sensitivity (Figure 4B). In the case of PaMoV, RT-PCR detection was effective up to 10^5^-fold dilution (6.93 × 10^−4^ ng/μL), while the RT-RAA-CRISPR/Cas12a methods remained reliable at a 10^7^-fold dilution (6.93 × 10^−6^ ng/μL), indicating a 10^2^-fold rise in sensitivity over conventional RT-PCR (Figure 4C).

### 2.5. The Development of the RT-RAA-CRISPR/Cas12a Detection System in Field Samples

**Figure 5:**
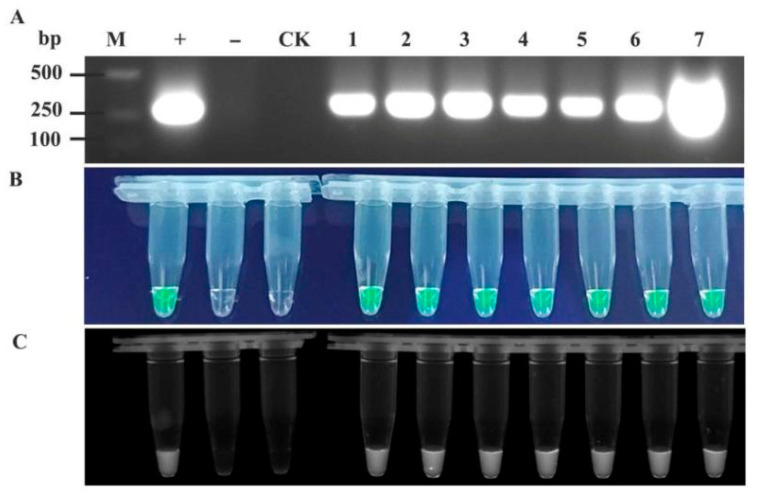
Comparison of the detection accuracy of TelMV in field samples by RT-RAA-CRISPR/Cas12a and RT-PCR. (**A**) RT-PCR detection; (**B**) detection using RT-RAA-CRISPR/Cas12a method (observed under blue light irradiation); and (**C**) detection using RT-RAA-CRISPR/Cas12a method (observed under UV transillumination). 1~7: Passionfruit leaf samples. “+” Positive control, “−” Negative control, “CK” Water control.

**Figure 6:**
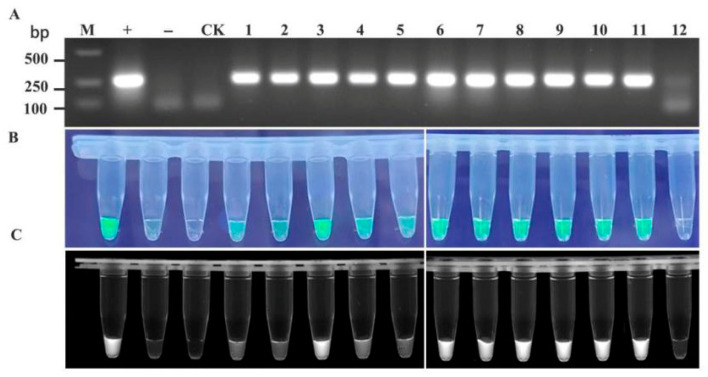
Comparison of the detection accuracy of EAPV in field samples by RT-RAA-CRISPR/Cas12a and RT-PCR. (**A**) RT-PCR detection; (**B**) detection using RT-RAA-CRISPR/Cas12a method (observed under blue light irradiation); and (**C**) detection using RT-RAA-CRISPR/Cas12a method (observed under UV transillumination). 1~12: Passionfruit leaf samples. “+” Positive control, “−” negative control, “CK” water control.

**Figure 7:**
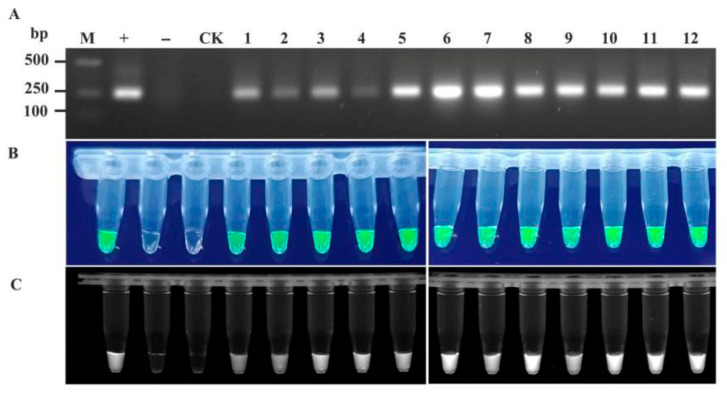
Comparison of the detection accuracy of PaMoV in field samples by RT-RAA-CRISPR/Cas12a and RT-PCR. (**A**) RT-PCR detection; (**B**) detection using RT-RAA-CRISPR/Cas12a method (observed under blue light irradiation); and (**C**) detection using RT-RAA-CRISPR/Cas12a method (observed under UV transillumination). 1~12: Passionfruit leaf samples. “+” Positive control, “−” negative control, “CK” water control.

To validate the feasibility of the RT-RAA-CRISPR/Cas12a detection system for TelMV, EAPV, and PaMoV, passionfruit samples with a suspected viral infection were collected from plantations in major passionfruit-producing regions of Guangxi (the cities of Nanning, Guilin, Wuzhou, Beihai, Qinzhou, Guigang, Yulin, and Baise). The samples were processed into crude extracts for RT-RAA-CRISPR/Cas12a detection, with RT-PCR results serving as controls. The two assay results were compared to identify discrepancies (partial results are shown in Figure 5, Figure 6 and Figure 7).

A statistical analysis was conducted on 199 disease samples collected from plantations in the main passionfruit production area in Guangxi using both RT-PCR and RT-RAA-CRISPR/Cas12a detection methods (Table 1). The results revealed that the three viral diseases in this area were relatively severe, and the detection rate when using the RT-RAA-CRISPR/Cas12a method was higher than that for RT-PCR. This indicated that the RT-RAA-CRISPR/Cas12a visual rapid detection method established in this study can be used for field rapid detection.

## 3. Discussion

In recent years, the passionfruit industry has experienced continuous growth, becoming a key driver of rural agricultural advancement. However, the widespread incidence of viral diseases has significantly hindered the safety of production. Early and rapid detection methods for viral pathogens detected are crucial for preventing and controlling the spread of these diseases. Currently, the diagnostic techniques for detecting TelMV, EAPV, and PaMoV in passionfruit primarily involve RT-PCR, RT-qPCR, and RT-LAMP, all of which necessitate laboratory conditions and specialized detection apparatus [6,25,26], and it takes a long time. Consequently, it is imperative to develop a rapid, sensitive, and visual detection method. Moreover, there are no documented rapid and visual detection methods available for identifying viral diseases in passionfruit.

In response to this gap, in the present study, we developed a visual detection method for RNA viruses utilizing the RT-RAA-CRISPR/LbCas12a system, which encompasses both RT-RAA reaction and CRISPR/LbCas12a detection. This method is both portable and cost-effective, enabling results to be directly observed with the naked eye under blue light. Consequently, it holds significant potential for field-based in situ detection. Compared to other plant virus detection methods, this technology offers notable advantages in three key areas: First, the RT-RAA-CRISPR/Cas12a detection system exhibits higher sensitivity; in our assessments, the RT-RAA-CRISPR/LbCas12a system exhibited a 10^2^-fold, 10^4^-fold, and 10^2^-fold increase in sensitivity for detecting TelMV, EAPV, and PaMoV, respectively, compared to direct RT-PCR methods. Second, the RT-RAA-CRISPR/Cas12a detection system exhibits higher time efficiency; whereas RT-PCRs typically requires at least one hour or more for completion, the RT-RAA-CRISPR/Cas12a fluorescence-based assay can produce results within 10~30 min. Consequently, the detection system enables in situ viral diagnosis to be achieved within approximately 30 min through straightforward sample collection and reagent addition. Third the RT-RAA-CRISPR/Cas12a detection system is characterized by its minimal equipment and instrument requirements. For this system, only a single-temperature water bath or a cup of warm water, and along with a UV flashlight. Unlike traditional PCR assays, the RAA reaction does not necessitate repeated thermal cycling, and the detection results are directly displayed via fluorescence within the reaction tube by the CRISPR/LbCas12a system.

To date, isothermal amplification in conjunction with the CRISPR/Cas12a nucleic acid recognition system has been extensively utilized to detect the detection of various viruses [27,28]. Importantly, the risk of aerosol contamination should not be underestimated during laboratory viral detection procedures. To mitigate the risk of false positives due to aerosol contamination from amplification product transfer, certain methodologies have been developed that integrating nucleic acid amplification and CRISPR detection within a single reaction tube have been developed [29,30]. Nevertheless, the sensitivity of these methods is generally compromised due to cross-interference between isothermal amplification reagents and the CRISPR/Cas12a detection system. The detection platform developed in this study is specifically tailored for field virus detection, where aerosol contamination is less common than in laboratory environments; consequently, a two-step approach is implemented to attain optimal detection sensitivity. In conclusion, a rapid and sensitive visual detection method for TelMV, EAPV, and PaMoV has been developed that exhibits high efficiency in detecting these viruses in passionfruit plants, with a detection limit markedly lower than that of RT-PCR. The system has been successfully applied in field detection, achieving detection rates that are comparable to or even exceed those obtained through laboratory-based RT-PCR.

## 4. Materials and Methods

### 4.1. Plant Samples

Passionfruit leaf samples exhibiting disease symptoms were collected from the field sites in Nanning City, Guangxi province, and the RNA extracts from TelMV-, EAPV-, and PaMoV-positive samples were confirmed as positive by RT-PCR and stored at −80 °C until further use.

### 4.2. RT-RAA Primer and crRNA Design and Synthesis

The whole-genome sequences of TelMV (MK340755.1 TeMV Wuyishan, ON932194.1 TeMV XW, MT557572.1 TeMV-GL2, MG944249.2 TelMV PasFru, DQ851493.1 TelMV Hanoi), PaMoV (MT990977.1 PfVNV NA1, NC_076243.1 PfVNV DaKNong, MK449340.1 PfSMoAV FJ), and EAPV (LC038071.1 EAPV SY144, LC325839.1 EAPV YW, AB690448.1 EAPV SY071) were downloaded from NCBI and aligned using MegAlign and Mega7.0. Specific primers and CrRNA probes (Table S1) were designed based on each virus’s conserved regions and synthesized by Sangon Biotech (Shanghai, China).

### 4.3. RT-RAA Assay

RT-RAA detection was performed using the RT-RAA kit (Cat: S003ZC, Hangzhou ZC Bio-Sci&Tech Co. Ltd., Hangzhou, China), while the reaction system was created following Wang et al. [31], simply comprising as 9.5 μL of buffer A, 9.5 μL of ddH_2_O, 1 μL of forward primer (10 μM), 1 μL of reverse primer (10 μM), 2 μL of buffer B, and 2 μL of RNA template. Buffer B was preloaded into the cap, and the reaction tube was rapidly centrifuged to ensure proper mixing of reagents, followed by undergoing 2% agarose gel electrophoresis. Total RNA was extracted from the leaf samples using the RNAiso Plus reagent (Cat: 9109, Takara, Dalian, China), while RT-PCR amplification was performed using the HiScript II One Step RT-PCR Kit (Cat: P612, Vazyme, Nanjing, China) with TelMV-, EAPV-, or PaMoV-specific RT-RAA primers. The reaction system (10 μL) contained 5 μL of 2× reaction mixture, 1 μL of RNA template, 0.25 μL of forward primer, 0.25 μL of reverse primer, and 3.5 μL of ddH_2_O. The RT-PCR detection procedure was as follows: 50 °C for 30 min; 95 °C for 5 min; 95 °C for 30 s, 55 °C for 30 s, and 72 °C for 30 s, for a total of 35 cycles; and 72 °C for 5 min. After amplification, the products were observed by 1% agarose gel electrophoresis.

### 4.4. The LbCas12a-Based Fluorescence Assay for Trans-Cleavage Activity

This protein was provided by Professor Zhang Tong, and for the collection and purification methods we referred to Wang et al. [31]. For the CRISPR/Cas12a reaction system, following Wang et al. [31], a total 20 μL reaction volume was used, consisting of 500 nM of LbCas12a, 500 nM of crRNA, 2 μL of 10× amplification buffer (100 mM of TrisHCl, 100 mM of sodium chloride, 150 mM of MgCl2, 10 mM of DTT, and 5% PEG-200), 2 μL of target gene plasmid, and 2 μM of fluorescent quencher-labeled ssDNA probe (5′-FAM-CCCCCCCCC-BHQ1-3′). The reaction was performed at 37 °C using the Varioskan LUX multi-functional microplate reader (Thermo, Waltham, MA, USA), with fluorescence measurements taken every 1 min (λex: 492 nm; λem: 520 nm). Fluorescence signals were also detected under a UV flashlight. To determine the lower detection limit of the CRISPR/Cas12a reaction, RT-RAA products were cloned into the pEASY-Blunt Zero vector (Cat: CB501, TransGen Biotech, Beijing, China) and the resulting plasmids were sequenced for verification.

To validate the feasibility of the CRISPR/Cas12a rapid detection system and the necessity of its components, and to further confirm the LbCas12a protein activity, the constructed pEASY^®^-Blunt Zero-TelMV, pEASY^®^-Blunt Zero-EAPV, and pEASY^®^-Blunt Zero-PaMoV positive standard plasmids were used as substrates for the detection system. Single-deletion treatments were performed on LbCas12a protein, crRNA, ssDNA reporter probe, 10× Enhanced Buffer, and target sequences, respectively. Using a multi-functional microplate reader, the reaction was carried out at 37 °C for 50 min, with fluorescence signal intensity monitored throughout (fluorescence intensity was measured every minute). After the reaction, the fluorescence status of the reaction tubes was observed under blue light irradiation.

### 4.5. Sensitivity and Specificity Assay

For the specificity assay, passionfruit leaf samples infected with different viruses were sampled for RT-RAA-CRISPR/Cas12a detection. For the sensitivity assay, total RNA was extracted from different virus-infected leaf samples and subjected to 10-fold serial dilutions (10^1^, 10^2^, 10^3^, 10^4^, 10^5^, 10^6^, and 10^7^) for RT-PCR and RT-RAACRISPR/Cas12a detection.

### 4.6. Field Sample Detection

To evaluate the CRISPR/Cas12a-based detection system, a total of 199 passionfruit leaf samples were systematically collected from diverse regions in Guangxi. For the RT-RAA reaction, they were incubated at 37 °C for approximately 30 min, and 2 µL of the RT-RAA product was used for the CRISPR/Cas12abased detection, as described above. Additionally, to validate the accuracy of the CRISPR/Cas12a-based detection system, the leaf samples were brought back to the laboratory and analyzed via RT-PCR.

## 5. Conclusions

In this study, we developed a visual detection system utilizing RT-RAA in conjunction with CRISPR/Cas12a for the detection of three viruses harmful to passionfruit production: TelMV, EAPV, and PaMoV. Subsequently, the system was applied using in-site detection of the three target viruses in field settings, yielding results that were fully consistent with laboratory-based RT-PCR assays. At present, the detection method is being developed into a portable detection kit for field testing of passionfruit, and we are currently seeking cooperative enterprises for commercial production. This method provides a more convenient technical tool for the field diagnosis and forecasting of viral diseases in passionfruit and holds substantial significance for the prevention and control of such diseases in China.

## Figures and Tables

**Table 1:** Statistical analysis of detection results for three viruses in the main production areas of passionfruit in Guangxi, based on two different detection methods: RT-PCR and RT-RAA-CRISPR/Cas12a.

Main Production Areas of Passionfruit	Total Samples	Infected Samples	Infection Rate (%) *	RT-PCR Detection Rate (%)			RT-RAA-CRISPR/Cas12a Detection Rate (%)		
				TelMV	EAPV	PaMoV	TelMV	EAPV	PaMoV
Nanning	18	15	83.3	83.3	44.4	27.7	88.9	44.4	27.8
Guilin	23	15	65.2	65.2	21.7	13	65.2	21.7	17.4
Wuzhou	16	14	87.5	81.3	62.5	62.5	81.3	62.5	62.5
Beihai	38	35	92.1	36.8	73.7	81.6	36.8	78.9	81.6
Qinzhou	35	17	48.6	14.3	8.6	37.1	14.3	8.6	37.1
Guigang	17	12	70.6	64.7	58.8	64.7	64.7	58.8	64.7
Yulin	34	31	91.2	88.2	47.1	23.5	88.2	52.9	23.5
Baise	18	18	100	94.4	61.1	88.9	94.4	61.1	88.9

* Infection rate(%) = The number of infected samples/total samples × 100.

## Data Availability

The original contributions presented in this study are included in the article/Supplementary Material. Further inquiries can be directed to the corresponding authors.

## Supplementary Materials

The following supporting information can be downloaded at: https://www.mdpi.com/article/10.3390/plants15060853/s1. Table S1: RAA primer used in this study.
