# Supplementary material for: In Situ Visual Detection of TelMV, EAPV, and PaMoV in Passionfruit Using Reverse Transcription-Recombinase-Aided Amplification and CRISPR/Cas12a
Source: Plants (Basel). 2026 Mar 10;15(6):853. doi: 10.3390/plants15060853 (PMC13029603; doi:10.3390/plants15060853)
Supplement: Supplementary file 1 [file plants-15-00853-s001.zip › plants-4152485-supplementary.pdf]

Supplementary Table S1. RAA primer used in this study.

Oligo names	Sequences (5' to 3')	Size
RAA-TelMV-1F	GGTCCCTCGGCAAACCAAGAAGAGTTCAAGCTA	296
RAA-TelMV-1R	GGATTCTTCCTTAAAAAGTATTTGTCATAACCG	
RAA-TelMV-2F	ACTGATAATCTTTCTTTGGGCACCTCTGTG	474
RAA-TelMV-2R	ATGAAGTCTTGCTCTATCTAGGCTCATTGG	
RAA-TelMV-3F	ATGCAAAGGTTGAATTTGTGCAGAGAGGTTC	269
RAA-TelMV-3R	CTTACCAATACGTCCCCTAACAATCATATAG	
RAA-TeMV-4F	GCATCGATGGTCTGGGCAAAGCTCAAGAACT	422
RAA-TeMV-4R	GGATTCTTCCTTAAAAAGTATTTGTCATAACCG	
RAA-TelMV-5F	GTCAAGCAACCAAAACTTAGCACAGTCTTTC	369
RAA-TelMV-5R	CTTACCAATACGTCCCCTAACAATCATATAG	
RAA-EAPV-1F	CATTGAGCCACACACTTGTACAGTTGATTTTGCG	260
RAA-EAPV-1R	CCCTTTGTTATCCGCAATACCAACTTCTCCACTAG	
RAA-EAPV-2F	GCCACACACTTGTACAGTTGATTTTGCGAATGC	253
RAA-EAPV-2R	CCCTTTGTTATCCGCAATACCAACTTCTCCACTAG	
RAA-EAPV-3F	GCCACACACTTGTACAGTTGATTTTGCGAATGC	292
RAA-EAPV-3R	AACCTGACTATCTCCATTGACGATTCAAGA	
RAA-EAPV-4F	CCTCTCTTGTAGGAAACATTTGCAGGCAATGAG	195
RAA-EAPV-4R	AACCTGACTATCTCCATTGACGATTCAAGA	
RAA-EAPV-5F	CCTCTCTTGTAGGAAACATTTGCAGGCAATGAG	309
RAA-EAPV-5R	ACTGTTTTGTTGCCTGATCCAGCTCTTGC	
RAA-PaMoV-1F	GTACCTCGCAATGCACATGGACTGTTGTGGACC	250bp
RAA-PaMoV-1R	ACGAGATGCCTTCTCAAGTTCATCCTGACTC	
RAA-PaMoV-2F	CTCAACTATCACAGACAGGGATTGCCTCAACCT	500
RAA-PaMoV-2R	GTGAAACGCCCAATGCGTTGTTGGTAGATGAT	
RAA-PaMoV-3F	GCGTTTCAAGACCAGACTGGGCGGAAAAGCTG	609
RAA-PaMoV-3R	GCGCGACAAGGCTAAACCAAAATTGTTGCCAAAAC	
RAA-PaMoV-4F	GACAATCGACATGCAATTACACGAGAAAACAC	227
RAA-PaMoV-4R	CGTATAATCGACGGTGCAGTTGTGTGAGTCCAC	
RAA-PaMoV-5F	AGTGGACTCACACAACTGCACCGTCGATTATAC	284
RAA-PaMoV-5R	GTGAAACGTCCAGTGCGTGGTTGGTGGTTGATGC	
TelMV-crRNA	UAAUUUCUACUAAGUGUAGAUGUCCAAGCAAUUGUUUGCAA	
EAPV-crRNA	UAAUUUCUACUAAGUGUAGAUGCACACUUAGAUUAUUGCAGCACC	
PaMoV-crRNA	UAAUUUCUACUAAGUGUAGAUCGCAACACACAUGGAAUGCU	
